# Interaction behavior of polyion-counterion for sodium polystyrene sulfonate

**DOI:** 10.1016/j.dib.2019.104365

**Published:** 2019-08-07

**Authors:** Ajaya Bhattarai

**Affiliations:** Department of Chemistry, M.M.A.M. C., Tribhuvan University, Biratnagar, Nepal

**Keywords:** Specific conductance, Sodium polystyrene sulfonate, Methanol–water mixture, Scaling theory approach, Counterion dissociation

## Abstract

The specific conductances are measured for sodium polystyrene sulfonate in water and methanol-water mixture. The equivalent conductivity data against concentration are evaluated by scaling concept. The effects of the solvent composition and the polyelectrolyte concentration on the fractions of uncondensed counterions (f), the polyion conductivities $\left(\lambda_{p}\right)$, the standard state free energies of counterion association $\left(\text{Δ}G_{A}^{o}\right)$, and the polyion transference number $\left(T_{P}\right)$ are calculated. The charge density (ξ) of sodium polystyrene sulfonate was used for the research article [1] to see the variation of the critical aggregation concentration of cetyltrimethyl ammonium bromide in sodium polystyrene sulfonate against the charge density of sodium polystyrene sulfonate.

**Table:** Specifications Table

Subject	Electrochemistry
Specific subject area	Polymer Chemistry
Type of data	Tables, figures
How data were acquired	Specific conductance data is measured from digital conductivity meter (Systronics India Ltd) and analysis of the data is performed by Q-Basic programming and easy plot software
Data format	Raw and analyzed data
Parameters for data collection	Specific conductance data were collected in excel file by varying the concentration of sodium polystyrene sulfonate by internal dilution method
Description of data collection	Specific conductance data of sodium polystyrene sulfonate were collected at 298.15 K in a thermos stated water-bath kept within ± 0.01 K
Data source location	Department of Chemistry, M. M.A.M.C., Tribhuvan University City: Biratnagar Country: Nepal
Data accessibility	Data is available with this publication
Related research article	A. Bhattarai, Micellization behavior of cetyltrimethylammonium bromide in the absence and presence of sodium polystyrene sulfonate in water and methanol-water mixture: A conductivity approach, Journal of Molecular Liquids. 292 (2019) 111352. https://doi.org/10.1016/j.molliq.2019.111352

**Table:** 

**Value of the Data** Investigated data highlight the evaluated equivalent conductivity (Λ) values against concentration (c) of sodium polystyrene sulfonate for determination of f which is a very important parameter for polyelectrolyte behavior. With the help of f and Λ; $\lambda_{\text{p}}$ can be calculated from the known values of $\lambda_{\text{c}}^{\text{0}}$ via equation $\left[\text{Λ}=f\left(\lambda_{\text{c}}^{\text{0}}+\lambda_{\text{p}}\right)\right]$. Here, $\lambda_{\text{c}}^{\text{0}}$ = counterion limiting equivalent conductivity. By using f, the values of the association constants ($K_{\text{A}}$) for the binding of the counterions onto to polyionic sites can be obtained from the equation [$\text{ln}K_{\text{A}}=\ln\left(\frac{1-f}{f}\right)-\text{ln}\left(fc\right)$] and $\text{Δ}G_{A}^{\text{o}}$ is further calculated as $\text{Δ}G_{A}^{\text{o}}=-RT\;\text{ln}K_{\text{A}}$ where R = Gas constant and *T* = temperature. The equivalent conductivity Λ) against the square root of sodium polystyrene sulfonate concentration √c ) in water and methanol-water mixture are perfectly matched with the scaling theory.

## 1 Data

The experimental specific conductance increases with the increase of concentration of NaPSS [2]. The experimental specific conductance data (Table 1) can be converted into equivalent conductance as the relation as

(1) $$\Lambda =0.001\left(\kappa -\kappa_{o}\right)/c$$

where κ = Experimental specific conductance of the solution.

**Table 1:** Specific conductance, κ of sodium polystyrene sulphonate in water and methanol-water mixtures at 298.15 K

Water		0.1 v.f.of methanol		0.2 v.f.of methanol	
c ×10^4^ equiv.l^−1^	κ μS/cm	c ×10^4^ equiv.l^−1^	κ μS/cm	c ×10^4^ equiv.l^−1^	κ μS/cm
12.11	89.79	12.25	70.60	13.32	58.12
10.04	76.05	10.18	59.86	11.09	49.44
08.41	65.03	08.47	50.94	09.24	42.08
06.97	55.12	07.07	43.53	07.67	35.75
05.80	46.94	05.90	37.17	06.40	30.54
04.84	40.11	04.93	31.92	05.33	26.09
02.89	25.86	04.08	27.13	04.45	22.37
01.69	16.69	02.89	20.30	03.72	19.24
0.810	09.63	01.69	13.26	02.89	15.62
		0.810	07.75	01.69	10.23
				0.810	06.08

$\kappa_{\text{o}}$= Experimental specific conductance of the solvent.

Λ= Equivalent conductance of the system.

By using Table 1 and Eq. (1), the equivalent conductance data is calculated. The equivalent conductance graphs are fitted with the help of scaling theory [3]:

(2) $$\Lambda =f\left[\lambda_{\text{c}}^{0}+\frac{Fz\text{e}f\text{c}\xi_{0}^{2}\;\ln\left(\xi_{0}/\xi_{e}\right)}{3\pi \eta_{0}}\right]$$

Here, F = Faraday number, Z = Counterion valence, e = Electronic charge, $\eta_{o}$ = Solvent viscosity coefficient, $\xi_{o}$ = Correlation blob size and $\xi_{e}$ = Electrostatic blobs size.

**Fig. 1:**
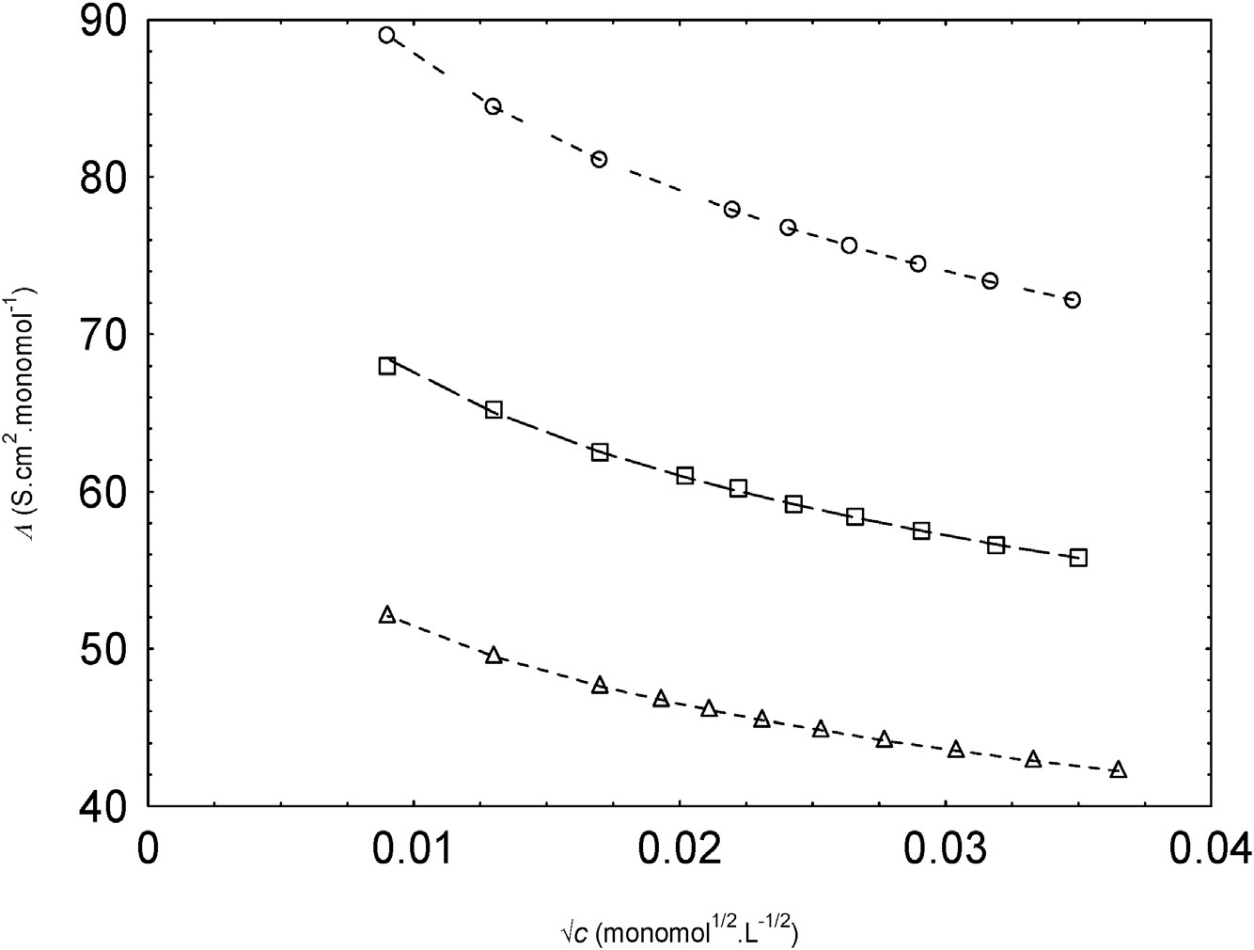
Variation of Λ with √c of NaPSS in water (circles), 0.1 vol fraction (v.f.) of methanol (squares) and 0.2 v.f. of methanol (triangles) at 298.15 K. Calculated values from scaling concept: dashed lines.

The relation (2) is used to the calculated Λ against c data for NaPSS to evaluate f which is a very important parameter for polyelectrolyte behavior. The experimental equivalent conductivity (Λ) against (√c ) for NaPSS in water and methanol-water mixture are perfectly matched with the scaling theory (Fig. 1). From this Fig. 1, it is clear that the equivalent conductivities show the increase with decreasing concentration within the concentration range investigated here. The experimental equivalent conductivities for NaPSS in water, 0.1 v.f. of methanol and 0.2 v.f. of methanol at 298.15 K are reported in Table 1S.

**Fig. 2:**
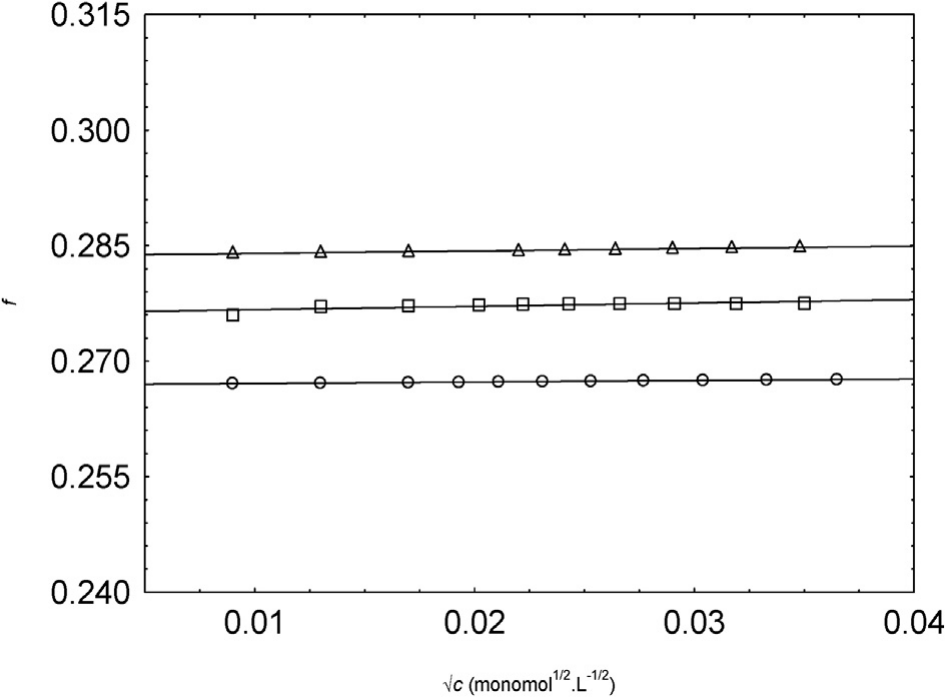
Variation of f with √c for NaPSS in water (triangles), 0.1 v. f. methanol (squares) and 0.2 v. f. methanol (circles).

Fig. 2 represents the plot of f vs. √c of NaPSS solutions. f values increase slightly as the concentration of NaPSS increases at 298.15 K. Such trends were also observed in the literature [2]. When the concentration of NaPSS increases, the relative permittivity of the system increases because of ionic polarizability of NaPSS [4]. Mohanty and Zhao in the year 1998 mentioned in their work that the increment of the effective dielectric constant of the system was not fully figured out [5], but the experimentally there is the evidence of the increment for the effective dielectric constant on the system [4], [6]. For NaPSS, especially, the frequency relied on dielectric constant is increased with the concentration of NaPSS [7]. Increment of dielectric constant (ε) of the system gives the reduction of Bjerrum length ($l_{\text{B}}=\frac{e^{2}}{\varepsilon K_{\text{B}}T}$). As $l_{B}$ settles the scale of the distance between the dissociated counterions on the poly-ion chain [4], [8], [9], there will be a small number of condensed counterions as the dielectric constant of the system rises. For charge density parameter, the structural value [10] for NaPSS in water was used (λ=2.83 at 298.15 K).

**Fig. 3:**
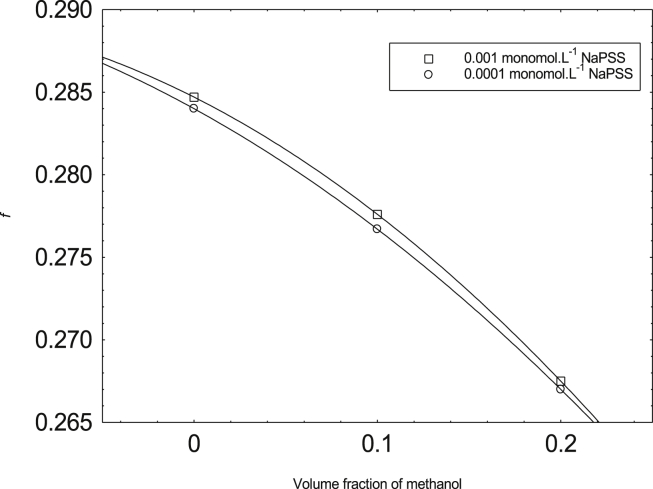
Variation of f with v. f. of methanol.

**Fig. 4:**
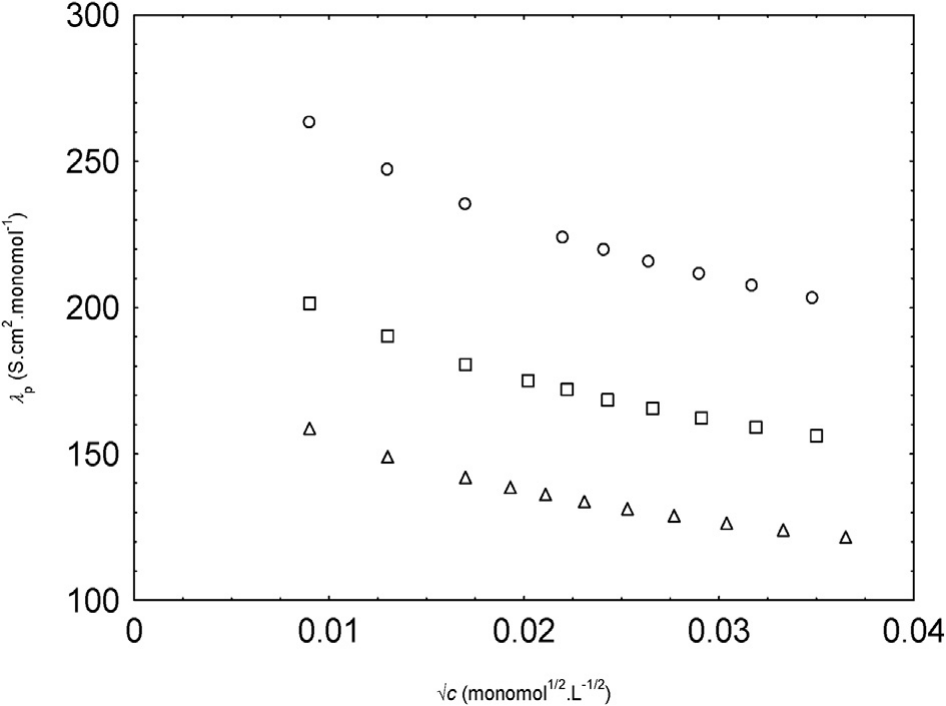
Variation of $\lambda_{{}_{P}}$ with √c for NaPSS in water (circles), 0.1 v.f. methanol (squares) and 0.2 v. f. methanol (triangles).

The observed f is reduced with the increment of methanol content of the mixture over the entire NaPSS concentration range investigated (Fig. 3). On increasing the methanol content, the dielectric constant of the system reduces at 298.15 K. Lower dielectric constant elevates higher counterion-binding and hence results in a lesser amount of uncondensed counterions in going water to 0.2 v. f. of methanol. Such trends were also seen in the same systems of higher temperatures [11] Usually, is given by [12]:

(3) $$\Lambda =f(\lambda_{\text{c}}^{\text{0}}+\lambda_{\text{p}})$$

where $\lambda_{\text{p}},$ f symbols have usual meanings. $\lambda_{\text{p}}$ can be calculated with the help of known values of Λ and f. The $\lambda_{\text{c}}^{\text{0}}$ values from taken from Table 2. The calculated values of $\lambda_{p}$ are plotted with the square root of NaPSS concentration (Fig. 4).

**Table 2:** Properties of water and methanol-water mixtures at 298.15 K and the corresponding $\lambda_{\text{c}}^{\text{0}}$ values of the Na ion with ξ calculated [$\xi =\frac{e^{2}}{b\varepsilon K_{\text{B}}T}$] with b = 2.52 A° and best-fitted ξ.

Calculated ( ξ) (Α°)	Best-fitted (ξ) (Α°)	$\eta_{0}$/mPa.s	ε	$\lambda_{\text{c}}^{\text{0}}$/S.cm^2^.mol^−1^
Water				
3.15	3.52	0.8959	78.30	50.10
0.1 v.f. of methanol				
3.29	3.61	1.1441	75.09	44.94
0.2 v.f. of methanol				
3.45	3.74	1.4346	71.61	36.78

Here $K_{B\;}$= Boltzmann constant.

The trends of the variation of $\lambda_{p}$ with √c of NaPPS (Fig. 4) were matched with the experimental equivalent conductivity of NaPSS in water and methanol-water mixture (Fig. 1).

To discuss the spontaneity of the counter-ion condensation procedure, information on $\text{Δ}G_{A}^{\text{o}}$ is necessary. For this aim, $K_{A}$ for the binding of the counterions onto to polyionic sites known as the equilibrium constant for the reaction can be obtained as:

Free site + ${\text{Na}}^{+}\Leftrightarrow$Combined site; has been estimated as a function of concentration from f taking the relation as:

(4) $$\text{ln}K_{\text{A}}=\ln\left(\frac{1-f}{f}\right)-\text{ln}\left(fc\right)$$

$\text{Δ}G_{A}^{\text{o}}$can very easily obtain from:

(5) $$\Delta G_{A}^{\text{o}}=-RT\;\text{ln}K_{\text{A}}$$

where R = gas constant and *T* = temperature.

**Fig. 5:**
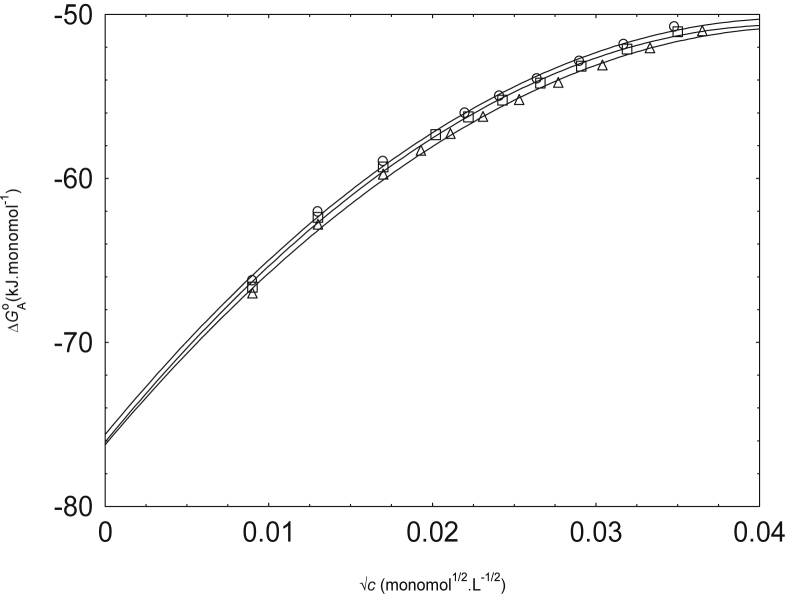
Variation of $\Delta G_{A}^{\text{o}}$ with √c for NaPSS in water (circles), 0.1 v. f. methanol (squares) and 0.2 v. f. methanol (triangles).

Fig. 5 displays the variation of $\text{Δ}G_{A}^{\text{o}}$ with √c of NaPSS in water and methanol-water mixtures. The negative $\text{Δ}G_{A}^{\text{o}}$ values suggest that the counterion condensation process is not nonspontaneous for NaPSS system over the full range of concentration even though the process becomes less spontaneous as the concentration rises. More increase of methanol to the system makes the counterion condensation procedure less suitable.

The transference number of the polyion ($T_{{}_{P}}$) has been calculated:

(6) $$T_{\text{P}}=\frac{\lambda_{\text{p}}}{\Lambda }$$

where the symbols have the usual meanings.

**Fig. 6:**
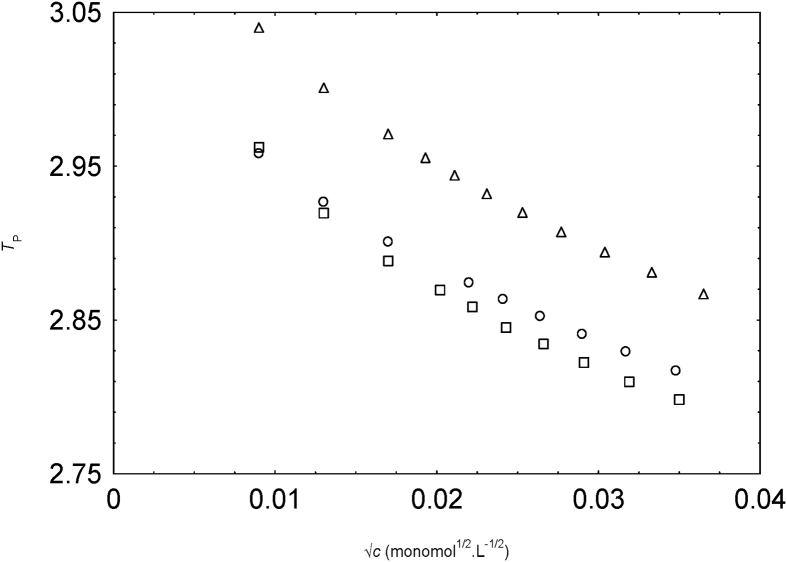
Variation of $T_{{\text{P}}_{}}$ with √c of NapSS ion in water (circles), 0.1 v. f. methanol (squares) and 0.2 v. f. methanol (triangles).

In Fig. 6, we present the plot of $T_{P}$ √c of NapSS in water and 0.1 and 0.2 v. f. methanol-water mixture at 298.15 K.$\;T_{\text{P}}$ is observed higher than 1 over the entire range of concentration in our study, and reduce with the increase of NaPSS concentration. The values of *T*_p_ were also higher than 1 for different aqueous polyions [13].

## 2 Experimental design, materials, and methods

Methanol was obtained from Merck, India. The purity of methanol is 99.0%. The binary mixture of water and methanol were made from 0.1 to 0.2 v. f. of methanol at 298.15 K constant temperature. Sodium polystyrene sulfonate (Sigma Aldrich, USA) of 70,000 g mol^−1^ with (1) a degree of sulfonation and (<1.2) a polydispersity index was purchased. The solution of sodium polystyrene sulfonate was made in triple distilled water and methanol-water mixture. The calibration of the conductivity cell was performed by 0.1 and 0.01 M KCl solutions [14]. The conductance was noted as described in the literature [1].
