# Supplementary material for: Interaction behavior of polyion-counterion for sodium polystyrene sulfonate
Source: Data Brief. 2019 Aug 7;25:104365. doi: 10.1016/j.dib.2019.104365 (PMC6710635; doi:10.1016/j.dib.2019.104365)
Supplement: Multimedia component 1 [file mmc1.docx]

Supplementary data for:

Interaction behavior of polyion-counterion for sodium polystyrene sulfonate

Ajaya Bhattarai

Department of Chemistry, M.M.A.M. C., Tribhuvan University, Biratnagar, Nepal

## Table 1S. Equivalent conductance, Λ of sodium polystyrene sulphonate in water and methanol-water mixtures at 298.15 K

| Water | | 0.1 v.f.of methanol | | 0.2 v.f.of methanol | |
| --- | --- | --- | --- | --- | --- |
| $c$ ×10^4^ *Λ*  equiv.l^-1^ S.cm^2^.equiv^-1^ | | $c$ ×10^4^ *Λ*  equiv.l^-1^ S.cm^2^.equiv^-1^ | | $c$ ×10^4^ *Λ*  equiv.l^-1^ S.cm^2^.equiv^-1^ | |
| 12.11  10.04  08.41  06.97  05.80  04.84  02.89  01.69  0.810 | 72.15  73.34  74.45  75.61  76.75  77.88  81.09  84.44  89.00 | 12.25  10.18  08.47  07.07  05.90  04.93  04.08  02.89  01.69  0.810 | 55.80  56.60  57.50  58.40  59.20  60.20  61.00  62.50  65.20  68.00 | 13.32  11.09  09.24  07.67  06.40  05.33  04.45  03.72  02.89  01.69  0.810 | 42.24  42.90  43.53  44.18  44.82  45.46  46.10  46.73  47.62  49.50  52.10 |
